# Acylated and Desacylated Ghrelin, Preptin, Leptin, and Nesfatin-1 Peptide Changes Related to the Body Mass Index

**DOI:** 10.1155/2013/236085

**Published:** 2013-11-25

**Authors:** Yusuf Ozkan, Esra Suay Timurkan, Suleyman Aydin, İbrahim Sahin, Mustafa Timurkan, Cihan Citil, Mehmet Kalayci, Musa Yilmaz, Aziz Aksoy, Zekiye Catak

**Affiliations:** ^1^Department of Endocrinology and Metabolism, Firat University Hospital, 23119 Elazig, Turkey; ^2^Department of Medical Biochemistry and Clinical Biochemistry, Firat Hormones Research Group, Firat University Hospital, 23119 Elazig, Turkey; ^3^Department of Histology and Embryology, Medical School, Erzincan University, 24100 Erzincan, Turkey; ^4^Atatürk Vocational School of Health Science, Kafkas University, 36040 Kars, Turkey; ^5^Department of Nutrition and Dietetic, Bitlis Eren University, 13000 Bitlis, Turkey

## Abstract

This study examines the levels of acylated and desacylated ghrelin, preptin, leptin, and nesfatin-1 peptide changes related to the body mass index (BMI). The subjects were allocated to 5 groups depending on their BMIs as follows: Group I (BMI <18.5 kg/m^2^); Group II (BMI 18.5–24.9 kg/m^2^); Group III (BMI 25–29.9 kg/m^2^); Group IV (BMI 30–39.9 kg/m^2^); Group V (BMI >40 kg/m^2^). Serum acylated and desacylated ghrelin, preptin, and leptin levels were measured by the enzyme-linked immunosorbent assay (ELISA) and nesfatin-1 was measured by the enzyme immunoassay (EIA). Desacylated ghrelin levels showed a gradual and statistically significant drop from Group I to Group V, while preptin and leptin levels exhibited a gradual and significant increase from Group I to Group IV. Serum nesfatin-1 levels gradually, but not significantly, increased from Group I to Group III and showed a significant decrease in Groups IV and V. In conclusion, leptin, preptin, and acylated ghrelin (AG) levels increased with higher BMI, whereas desacylated ghrelin (DAG) decreased and nesfatin-1 showed no clear relationship to BMI.

## 1. Introduction

Obesity is becoming increasingly prevalent throughout the world, particularly in developed countries. The precise etiology of obesity remains obscure. However, orexigenic (acylated and desacylated ghrelin and preptin) and anorexigenic (nesfatin-1 and leptin) peptide hormones of the endocrine system may play a critical role in the development of obesity by regulating the energy balance and affecting the “eating center” and “satiety center” in the paraventricular nucleus, arcuate nucleus, and nucleus of the solitary tract.

Of the orexigenic peptides, ghrelin was discovered in 1999 by Kojima et al. [1] in the X/A cells of the fundus and pylorus areas of the rat stomach and P/D1 cells of the stomach in humans. Ghrelin, which contains 28 amino acids, is called acylated ghrelin when it has a fatty acid attached to the N terminus of the third serine (octanoic acid), and is called desacylated ghrelin when it has no fatty acid attached [2, 3]. Both forms of ghrelin are present in tissues and biological fluids [4]. The effect of desacylated ghrelin on food intake in mice is weaker than acylated ghrelin. Behavioral studies led to controversial results regarding desacylated ghrelin, and albeit several studies suggest that it is anorexigenic peptide counteracting some of AGs actions [5]. Ghrelin levels in humans decrease with obesity and calorie intake and increase with hunger and in anorexia nervosa patients [6].

Preptin is a 34-amino acid peptide hormone cosecreted from the *β* cells of pancreas along with insulin, amylin, and pancreastatin [7, 8]. Its precursor is Pro-IGF-II, which also produces insulin-like growth factor II (IGF-II). IGF-II is involved in the regulation of cell growth, differentiation, and metabolism [9]. Females have higher preptin levels than males [9]. Preptin is believed to be a physiological enhancer of insulin secretion induced by glucose. There is a strong correlation between obesity and hyperinsulinemia and insulin resistance, and these get stronger with increasing body weight [10]. Therefore, the relationship between BMI and preptin level would be worth investigation.

Nesfatin-1, a recently found anorexigenic peptide, is derived from the 82-amino acid protein called nucleobindin-2 (NUCB2), known as a satiety molecule in the hypothalamus [11, 12]. The middle segment of nesfatin-1 (residues 24 to 53) is biologically active, and its injection reduces food intake, whereas the N-23 and the C-29 amino acid terminal segments are inactive. Nesfatin-1 is a new anorexigenic factor and energy stabilizer [13, reviewed]. Shown to reduce food intake, nesfatin-1 has an inhibitory effect on food intake and thus alleviates obesity in a dose- and time-dependent manner upon intracerebroventricular and intraperitoneal injection, as well as after intranasal administration [14]. It is also known that there is a slightly positive but inconsistent correlation of nesfatin-1 with BMI in humans, since variables as sex and stress seem to confound this relationship [13, reviewed].

 Leptin, an anorexigenic agent, is a polypeptide hormone containing 167 amino acids. Leptin is secreted primarily by adipocytes in the fat tissue. Serum leptin levels correlate with body mass index (BMI), serving as the key regulator of body weight through its neuroendocrine functioning [15]. Leptin is bound in low-weight (thin) individuals but is free in obese individuals [16], its rhythmic release being affected by the time of eating, with lowest level in the morning and peaking at night [17]. Leptin levels are also affected by sex, being higher in women than in men [18]. Since it is involved in the hunger and satiety centers of the brain, it functions primarily as a metabolic signal associated with the sufficiency—rather than a surplus—of energy. Its signal when reaching the hypothalamus brings the indication that fat reserves are full and thus inhibit expression of orexigenic peptides, neuropeptide Y in particular, while increasing the expression of anorexigenic peptides, thereby decreasing food intake and increasing energy consumption [19, 20].

In the light of these data, this study investigated cross-sectional in five BMI groups from underweight to morbidly obese subjects the serum levels of several peptides involved in the regulation of hunger and satiety: ghrelin in its acylated and desacylated form, nesfatin-1, leptin, and the physiologic amplifier of glucose-mediated insulin secretion preptin.It was found that leptin, preptin, and acylated ghrelin (AG) levels increased with higher BMI, whereas desacylated ghrelin (DAG) decreased and nesfatin-1 showed no clear relationship with BMI.

## 2. Material and Method

### 2.1. Selection of Patients and Collection of Samples

The study was carried out in the Internal Medicine Department and Biochemistry Department of Firat University, School of Medicine. After the approval of the Ethics Board had been obtained, the patients were informed about the study and written consents obtained. Registered individuals ranging from 18 to 70 in age study were allocated to one of 5 groups depending on their BMIs. In order to assess subjective appetite sensations, visual analogue scale (VAS) was applied. Subjects who had similar visual analogue scale (VAS) were included. Classification of groups by their BMIs was as follows: Group I (BMI <18.5 kg/m²—low-weight); Group II (BMI 18.5–24.9 kg/m²—normal-weight); Group III (BMI: 25–29.9 kg/m²—overweight); Group IV: (BMI 30–39.9 kg/m²—obese); Group V: (BMI >40 kg/m²—morbidly obese). Insulin resistance was calculated using the homoeostasis model assessment of insulin resistance (HOMA-IR) index (fasting insulin (units per milliliter) * fasting glucose (millimolar)/22.5).

BMI (kg/m^2^) was calculated by using body weight (kg) and the height in square meters (m^2^). None of the subjects had diabetes type 1 or 2 (including the family history), moderate to severe hypertension (resting blood pressure (BP) >130/80–>140/85 mmHg), acute infectious disease, and chronic medical illness. Pregnant women and subjects who had gastrointestinal disease were excluded and anyone using use drugs, tobacco products (former and current), and alcoholic drinks. Those doing intense exercise regularly (>15 min of aerobics 3 times per week) were also excluded. None of the subjects had undergone gastrointestinal surgery.

To conduct biochemical analyses, venous blood samples (8 mL) were collected from the patients after 12 h of fasting. Four mL was taken for routine biochemical parameters. The remaining 4 mL was put into tubes containing 500 Kallikrein Inhibitor Unit (KIU) aprotinin and centrifuged at 4000 rpm (1792 ×g) for 5 min. Serum samples from the centrifuging process were transferred to Eppendorf tubes and stored at −20°C until analyzed [21].

### 2.2. Laboratory Analyses

Blood glucose, total cholesterol, low-density lipoprotein (LDL), LDL-cholesterol, high-density lipoprotein (HDL), HDL-cholesterol, very low-density lipoprotein (VLDL), VLDL-cholesterol, triglyceride (TG), urea, creatine, aspartate aminotransferase (AST), and alanine aminotransferase (ALT) were analyzed using the Olympus AU 600 autoanalyzer. Whole blood count was measured in CELL-DYN 3700 blood count device. Thyroid function (serum triiodothyronine (sT3), total serum thyroxin (sT4), thyroid stimulating hormone (TSH), adrenocorticotropic hormone (ACTH), cortisol, insulin, and C-peptide) was assessed using the Immulite 200 device and HbA1c Shimadzu Silver 20A Prominence apparatus.

Serum acylated ghrelin levels were analyzed using human acylated ghrelin ELISA commercial kit (Cat. No: A05106 SPI-BIO, Human Acylated Ghrelin Enzyme Immunoassay Kit, France), and desacylated ghrelin was measured using human unacylated ghrelin ELISA commercial kit (Cat. no. A05119, SPI-BIO, Human Unacylated Ghrelin Immunoassay Kit) in an ELX 800 ELISA reader. Serum preptin was measured with the human preptin ELISA kit (Cat. no. CSB-E09772 h, CUSABIO BIOTECH Co. LTD.), serum nesfatin-1 with Human/Mouse/Rat Nesfatin Enzyme Immunoassay Kit (Cat. no. EIA-NES-1, RayBiotech, Inc.), and serum leptin with Leptin (Sandwich) ELISA kit (Cat. no. EIA-2395, DRG Instruments GmBh). The values were read at 410 nm and 450 nm wavelength spectrophotometrically. The results were calculated by multiplying the values at the dilution rate.

### 2.3. Statistical Analyses

Statistical analyses were conducted using the SPSS 12.0 for Windows package software. Nonparametric statistics was applied. Possible differences between the parametric data of groups used ANOVA and Post Hoc Tukey Test, when necessary, while possible correlation among parameters used the Pearson's correlation analysis method. After adjusting age and BMI, Pearson's and Spearman's correlation coefficients with a Bonferroni correction were calculated to examine the relationship between orexigenic and anorexigenic peptides and obesity. Statistical significance was set at *P* < 0.05. The data are given as mean ± standard deviation.

## 3. Results

The comparison of the groups in terms of their demographic characteristics showed a statistically significant difference between the mean age and BMI values of the groups (*P* < 0.001). The highest mean age among the groups was in group III (55.2 ± 12.8) and the lowest mean age in Group I (28.8 ± 16.2). The significantly lowest BMI occurred in group I (17.6 ± 0.8); in the other groups BMIs were in ascending order from Group II (21.7 ± 1.6), Group III (27.4 ± 2.1), Group IV (34.9 ± 2.46), to Group V (44.8 ± 4.2). Demographic characteristics and routine biochemical parameters of the patients are given in Table 1. Fasting glucose levels increase from underweight to overweight and then are lower when measured in different states of obesity. Additionally, insulin, HOMA-IR, total cholesterol, LDL, and triglycerides are higher in Group II, III, or IV than those of morbidly obese subjects (Table 1). The cause of such fluctuations is currently not known. However, it is possible that comorbidities and medication may play a role in the fluctuations of these parameters.

**Figure 1:**
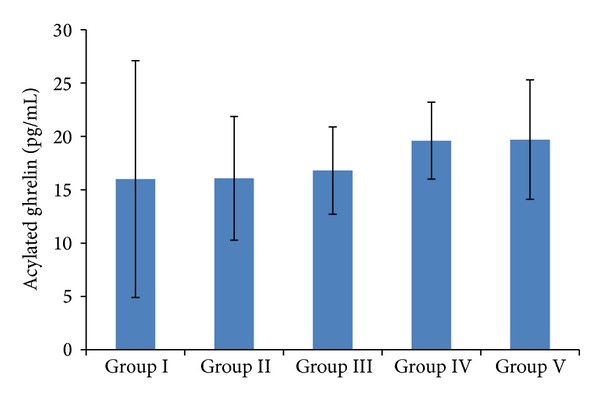
Serum acylated ghrelin levels in relation to BMIs. Group I: low-weight; Group II: normal-weight; Group III: overweight; Group IV: obese; Group V: morbidly obese. *P* < 0.005 for Group I versus Group IV and Group V.

 Serum acylated ghrelin levels of the participants by BMI were 16.0 ± 11.1 in Group I, 16.07 ± 5.8 in Group II, 16.8 ± 4.1 in Group III, 19.6 ± 3.6 in Group IV, and 19.7 ± 5.6 pg/mL in Group V (Figure 1). When the groups were compared individually with each other, serum acylated ghrelin levels followed an ascending order from Group I to Group V; that is, they were the lowest in low-weight (thin) individuals and the highest in obese individuals (Figure 1).

**Figure 2:**
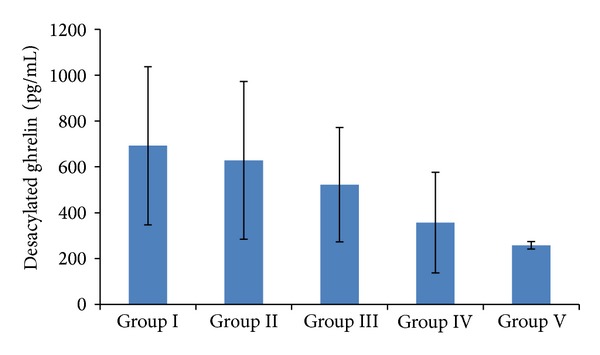
Serum desacylated ghrelin levels in relation to BMIs. Group I: low-weight; Group II: normal-weight; Group III: overweight; Group IV: obese; Group V: morbidly obese. *P* < 0.005 for Group I versus Group II versus Group III versus Group IV and Group V.

Serum desacylated ghrelin levels (Figure 2) were 692 ± 345 in Group I, 628.4 ± 344.2 pg/mL in Group II, 522.0 ± 249.5 in Group III, 356.8 ± 219.4 in Group IV, and 257.7 ± 16.1 pg/mL in Group V. Comparison of groups with each other showed that serum desacylated ghrelin levels decreased with increasing BMI. The highest desacylated ghrelin levels were in low-weight (thin) individuals and the lowest in morbidly obese individuals. In normal-weight individuals, the levels were significantly lower than in obese and morbidly obese individuals (*P* < 0.01, *P* < 0.001, resp.) (Figure 2).

**Figure 3:**
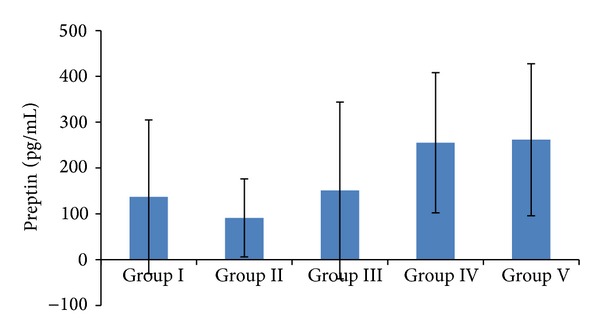
Serum preptin levels in relation to BMIs. Group I: low-weight; Group II: normal-weight; Group III: overweight; Group IV: obese; Group V: morbidly obese. *P* < 0.005 for Group I versus Group II versus Group III versus Group IV and Group V.

Serum preptin levels were the lowest in the normal-weight individuals of Group II. They were 137 ± 168 in Group I, 91 ± 85 in Group II, 151 ± 193 in Group III, 255 ± 153 in Group IV, and 262 ± 166 pg/mL in Group V. Preptin levels in low-weight and overweight individuals were slightly higher than in normal-weight individuals. Obese and morbidly obese groups had significantly higher preptin levels than low-weight and overweight individuals (*P* < 0.001) (Figure 3).

**Figure 4:**
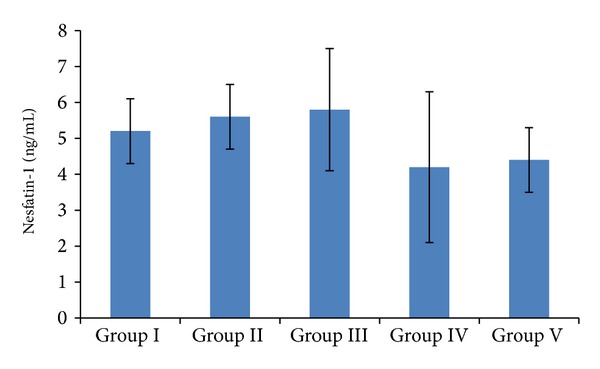
Serum nesfatin-1 levels in relation to BMIs. Group I: low-weight; Group II: normal-weight; Group III: overweight; Group IV: obese; Group V: morbidly obese. *P* < 0.005 for Group I versus Group IV and Group V.

Serum nesfatin-1 levels (Figure 4) were 5.2 ± 0.9 in Group I, 5.6 ± 0.9 in Group II, 5.8 ± 1.7 in Group III, 4.2 ± 2.1 in Group IV, and 4.4 ± 0.9 ng/mL in Group V. Comparing each group with the others, levels followed the following ascending order: Group IV < Group V < Group I < Group II < Group III. Comparison of group II with Groups IV and V showed that Group II had significantly higher serum nesfatin-1 levels than the other 2 groups (*P* < 0.01, *P* < 0.05, resp.). Similarly, comparing nesfatin-1 levels of Group III with those in Groups IV and V, significantly elevated levels were present in Group III (*P* < 0.001, *P* < 0.01, resp.) (Figure 4).

**Figure 5:**
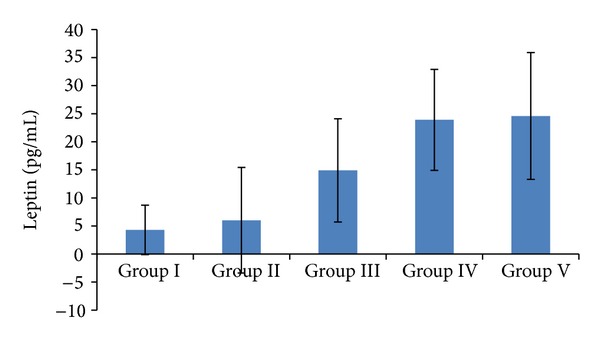
Serum leptin levels in relation to BMIs. Group I: low-weight; Group II: normal-weight; Group III: overweight; Group IV: obese; Group V: morbidly obese. *P* < 0.005 for Group I versus Group II versus Group III versus Group IV and Group V.

Serum leptin levels were the lowest in low-weight individuals: 4.3 ± 4.4 in Group I, 6.0 ± 3.94 in Group II, 14.9 ± 4.92 in Group III, 23.9 ± 9.0 in Group IV, and 24.6 ± 11.3 pg/mL in Group V. Leptin levels were higher in the individuals who had high BMIs. Overweight individuals had statistically significantly higher leptin levels than low- and normal-weight individuals (*P* < 0.001, *P* < 0.01). Leptin levels in the obese group were higher than those in low-weight, normal-weight and overweight individuals, the differences being statistically significant (*P* < 0.001, *P* < 0.001, *P* < 0.01; Figure 5). Correlations found between acylated and desacylated ghrelin, preptin, nesfatin-1, and leptin levels and BMI are given in Table 2. Based on age, there were also correlations in Groups II and IV (Table 2).

## 4. Discussion

Obesity, which is becoming increasingly widespread around the world, is estimated to have a prevalence of 8.2%. When obesity was accepted as a BMI ≥30 kg/m^2^, US studies between 1982 and 1984, and between 1988 and 1998, showed that obesity increased from 16.5 to 25% in females and from 12 to 20% in males. These rather high figures are similar to those in almost all developed and developing countries of the world [22]. A study was conducted in Turkey in 1996 as the first of its kind; the prevalence of obesity was 18.6%, a figure that rose to 21.9% in 2002 [23, 24]. Thus, the prevalence of obesity in Turkey was not different from that in the developed countries of the western world, and it reached markedly high levels particularly in females, among whom the prevalence of obesity was as high as 30% [25].

Obesity has a greater effect on ALT than on AST. In line with the results of our study concerning the relation between ALT and obesity, we found that 16% of the individuals who had a BMI of 30 kg/m^2^ or above had higher ALT levels, consistent with Bruckert et al. [26] and Clark et al. [27]. Serum lipid levels also rose with increasing BMI from Group I to Group IV, being statistically significant in all groups. Obesity develops when the chronic mismatch between energy intake and consumption produces a surplus of energy, which is stored as excess fat in the form of TG in the adipose tissue [28]. In a normal human being, the rate of feeding is reduced to prevent excessive storage once fat and carbohydrate reserves exceed the optimal level.

Previous studies showed that plasma ghrelin levels were higher in cachexia and lower in obesity [29, 30]. Plasma ghrelin level is inversely related to BMI. It increased with weight loss and decreased when the lost weight is regained [31]. Ghrelin levels in obese individuals are lower [32], and Sahin et al. [33] reported reduced serum ghrelin levels in the gastrointestinal system tissues and serum of rats, which were obese due to diet. We think that the decrease in desacylated ghrelin levels in our study resulted from elevated glucose levels in obesity. Desacylated ghrelin levels are known to decrease with increasing glucose and increase with decreasing glucose [34]. Ghrelin levels are reported to be decreased in obesity [35]. However, surprisingly, active acylated ghrelin levels in our analysis were elevated in line with increased BMI, although the increase was not statistically significant. Phoenix Pharmaceuticals Belmont, CA company ghrelin kits measure total ghrelin, not specifically acylated ghrelin. Increased acylated ghrelin concentrations observed in obesity might represent a physiological adaptation to the regulation of energy balance associated with obesity. Collectively, these data suggest why acylated ghrelin increased with BMI, which needs to be further investigated.

Serum desacylated ghrelin levels, on the other hand, decreased with increasing BMI. The decrease in serum desacylated ghrelin levels in obese and morbid obese individuals was statistically significant compared to lower level in normal-weight participants. Leptin and insulin have been shown to be peripheral regulators of ghrelin secretion, and a reciprocal relationship exists between plasma ghrelin and leptin or insulin levels [36]. Increased leptin might cause a decrement of desacylated ghrelin beside acylated ghrelin as reported here. In this study indeed, desacylated ghrelin levels are decreased in human obesity, whereas leptin levels are increased, and the effects of desacylated ghrelin on energy homeostasis are opposite to those of leptin and acylated ghrelin.

Also, it was first time shown that serum preptin concentrations increased with increasing BMI. However, we could not find any report to compare the effects of preptin levels and appetite and BMI in obese individuals. Yang et al. [37], reported that the circulating level of preptin was 398 ± 13 ng/L in normal-weight individuals, with levels in males being lower than in females. Their study showed a positive correlation between plasma preptin level and diastolic blood pressure, TG, total cholesterol, HbA1c, and HOMA-IR index. Our preptin levels rose with increasing BMI, as in Yang et al. [37], but the increase was not statistically significant.

Nesfatin-1 levels increased from low-weight individuals to those who were overweight. Ramanjaneya et al. [38] found that nesfatin-1 was a depot-specific adipokine produced preferably by the adipose tissue in obese individuals and individuals with arranged dietary deprivation. Nesfatin-1 levels were statistically higher in mice fed on a high-fat diet (*P* < 0.05) and correlated positively with human BMI (*P* < 0.01). They fell in food-deprived individuals compared to the control group (*P* < 0.01). Tsuchiya et al. [39] prepared especially sensitive ELISA to measure nesfatin-1 level after oral glucose tolerance test (OGTT) and food tests following overnight fasting. There were 43 nonobese males (age: 24.5 ± 0.3; BMI: 21.2 ± 0.3 kg/m^2^) and 9 males with a high BMI (age: 32 ± 3.7; BMI: 37.3 ± 3.8 kg/m^2^), all of whom were given 75 g OGTT and a food test before their fasting nesfatin-1 concentrations were quantified. The concentrations showed a significant negative correlation with BMI, body fat percentage, body fat weight, and blood glucose (*P* < 0.05). Nesfatin-1 correlations did not change significantly during OGTT and food test. However, fasting plasma nesfatin-1 levels were significantly lower in the individuals with a high BMI than those with a normal BMI. Nesfatin-1 decreased with increasing body weight and functioned as a new anorexigenic factor and energy stabilizer [40]. Reduction in the blood nesfatin-1 level with rising BMI might be related to impaired insulin sensitivity and glucose homeostasis.

In our groups, leptin levels also showed positive correlation with BMI in Groups II and III. Nakahara et al. [41] compared plasma ghrelin and leptin responses to exogenous and endogenous stimuli in intact rats and other that had been starved for 16 h to inhibit the increase in ghrelin secretion due to hunger. High insulin levels led to higher leptin levels [42]. 

Additionally, high and low ambient temperatures, stress, or insulin supplementation affected plasma ghrelin [43]. Bell-Anderson and Bryson [44] successfully used leptin in the treatment of leptin-deficient obese patients and consequently determined that leptin levels were associated with BMI.

## 5. Conclusion

As BMI increases, desacylated ghrelin decreases. Preptin and leptin levels, however, rise with increasing BMI. Also, acylated ghrelin rose in obesity and nesfatin-1 showed no clear relationship to BMI. These peptides have “crosstalk” in their etiopathogenesis.

## Figures and Tables

**Table 1:** Demographic characteristics and routine biochemical parameters of study groups.

Parameters	Group I	Group II	Group III	Group IV	Group V
	(*n*: 31)	(*n*: 28)	(*n*: 31)	(*n*: 30)	(*n*: 30)
	(16 F, 15 M)	(14 F, 14 M)	(16 F, 15 M)	(15 F, 15 M)	(15 F, 15 M)
Age (years)	28.8 ± 16.2	40.8 ± 20.4^a^	55.2 ± 12.8^a,c^	52.06 ± 15.2^c^	45.8 ± 12.8^c^
BMI (kg/m^2^)	17.6 ± 0.8	21.7 ± 1.6^c^	27.4 ± 2.1^c^	34.9 ± 2.46^c^	44.8 ± 4.2^c^
Glucose (mg/dL) in hunger	89.8 ± 33.2	113.6 ± 7.2.^b^	202.07 ± 9.9^a,c^	192.2 ± 29.6^c^	149.9 ± 9.7
Insulin (*μ*U/mL) in hunger	14.1 ± 11.2	22.4 ± 3.7	20.3 ± 2.4	16.5 ± 8.8	19.7 ± 1.8
HOMA-IR	3.3 ± 3.1	6.9 ± 10.5	9.8 ± 11.1	7.9 ± 7.7	8.4 ± 12.0
Total cholesterol (mg/dL)	159.7 ± 44.2	177.8 ± 44.8	207.0 ± 61.5^b^	210.3 ± 52.7^c^	184.7 ± 42.6
LDL (mg/dL)	93.0 ± 33.3	112.8 ± 37.1	133.7 ± 41.4^c^	139.0 ± 37.3^c^	122.3 ± 34.7^a^
HDL (mg/dL)	48.4 ± 14.5	52.9 ± 15.2	49.4 ± 18.8	52.2 ± 22.5	50.06 ± 17.5
VLDL (mg/dL)	17.2 ± 10.1	20.1 ± 9.5	42.8 ± 4.6	43.9 ± 3.2	37.8 ± 2.2
TG (mg/dL)	91.9 ± 5.5	106.6 ± 5.2^a^	208.0 ± 22.8.4^a^	219.7 ± 17.04^a,b^	182 ± 11.9
AST (U/l)	22.6 ± 10.8	21.9 ± 7.2	21 ± 5.5	30.3 ± 3.9	25.7 ± 1.7
ALT (U/l)	16.5 ± 6.5	19.9 ± 1.8	22.1 ± 5.5	31.2 ± 3.2	37 ± 51.9^a^

^a^ *P* < 0.05: age Groups I→II, age Groups II→III, ALT Groups I→V, glucose Groups II→III, LDL Groups I→V, TG Groups I→III, and TG Groups II→IV.

^b^ *P* < 0.01: glucose Groups II→III, TG Groups I→IV, and Total cholesterol Groups I→III.

^c^ *P* < 0.001: age Groups I→III, age Groups I→IV, age Groups I→V, BMI Groups I→II, BMI Groups I→III, BMI Groups I→IV, BMI Groups I→V, BMI Groups II→III, BMI Groups II→IV, BMI Groups II→V, BMI Groups III→IV, BMI Groups III→V, BMI Groups IV→V, Glucose Groups I→III, Glucose Groups I→IV, LDL Groups I→III, LDL Groups I→IV, and total cholesterol Groups I→IV.

**Table 2:** Correlation between acylated ghrelin, desacylated ghrelin, preptin, nesfatin-1, and leptin levels and BMI in study groups.

Peptides	Group I		Group II		Group III		Group VI		Group V	
	(*n*: 31)		(*n*: 28)		(*n*: 31)		(*n*: 30)		(*n*: 30)	
	(16 females, 15 males)		(14 females, 14 males)		(16 females, 15 males)		(15 females, 15 males)		(15 females, 15 males)	
	*r*	*P*	*r*	*P*	*r*	*P*	*r*	*P*	*r*	*P*
Acylated ghrelin	−0.563	0.001	−0.071	0.721	0.147	0.438	−0.213	0.258	0.031	0.870
Desacylated ghrelin	−0.174	0.349	0.032	0.870	0.123	0.519	0.099	0.603	−0.125	0.509
Preptin	0.194	0.296	0.190	0.332	−0.144	0.447	−0.400	0.029	0.164	0.385
Nesfatin-1	0.111	0.551	−0.370	0.853	0.180	0.342	−0.760	0.688	−0.149	0.433
Leptin	−0.001	0.995	0.109	0.582	0.220	0.243	−0.111	0.561	−0.208	0.270

In brief, the increase in serum preptin and leptin levels is in a positive correlation with BMI. However, a fluctuation was observed in nesfatin-1 levels in such a way that nesfatin-1 levels increased in individuals with a BMI of up to 30 kg/m^2^ but decreased in obese (Group IV) and morbidly obese (Group V) individuals. Desacylated ghrelin dropped in all groups with increasing BMI that is, desacylated ghrelin is inversely correlated with BMI, while acylated ghrelin fluctuated.

## Abbreviations

ACTH: Adrenocorticotropic hormone
ALT: Alanine aminotransferase
AST: Aspartate aminotransferase
BMI: Body mass index
BP: Blood pressure
HDL: High-density lipoprotein
HOMA-IR: Homoeostasis model assessment of insulin resistance ()
IGF-II: Insulin-like growth factor II
KI: Kallikrein inhibitor unit
LDL: Low-density lipoprotein
NUCB2: Nucleobindin-2
OGTT: Oral glucose tolerance test
sT3: Serum triiodothyronine
sT4: Serum thyroxin
TG: Triglyceride
TSH: Thyroid stimulating hormone
VAS: Visual analogue scale
VLDL: Very low-density lipoprotein.
